# A novel multi‐channel applicator with a U‐shaped channel for vaginal intracavity brachytherapy

**DOI:** 10.1002/acm2.14521

**Published:** 2024-09-11

**Authors:** Yanfei Lu, Jianhong Chen, Fangfang Wang, Qiong Zhou, Xiang Zhang

**Affiliations:** ^1^ Zhejiang Cancer Hospital Hangzhou Zhejiang China; ^2^ Hangzhou Institute of Medicine (HIM) Chinese Academy of Sciences Hangzhou Zhejiang China

**Keywords:** applicator, brachytherapy, dose distribution, endometrial cancer

## Abstract

**Background:**

Endometrial cancer is one of the most common gynecological malignancies in the world. Vaginal brachytherapy is an important postoperative adjuvant treatment for endometrial cancer. However, a common problem with existing applicators is insufficient dose at the vaginal apex.

**Purpose:**

This study describes the Hangzhou (HZ) cylinder, a novel 3D printed vaginal intracavity brachytherapy applicator, detailing its characteristics, dose distribution, and clinical applications.

**Methods and Materials:**

The HZ cylinder is distinguished by its unique structure: a U‐shaped channel with a 2 mm diameter, a straight central axis channel of the same diameter, and 10 parallel straight channels. For comparison, standard plans were employed, designed to ensure that a minimum of 95% of the prescribed dose reached 5 mm beneath the mucosal surface. We conducted comparative analyses of mucosal surface doses and doses at a 5 mm depth below the mucosa between the HZ cylinder and a conventional single‐channel cylinder across various treatment schemes. Additionally, the study examined dose differences in target volume and organs at risk (OARs) between actual HZ cylinder plans and hypothetical single‐channel plans.

**Results:**

In the standard plans, mucosal surface doses at the apex of the vagina were 209.32% and 200.61% of the prescribed dose with the HZ and single‐channel cylinders, respectively. The doses on the left and right wall mucosal surfaces varied from 149.26% to 178.13% and 142.98% to 180.75% of the prescribed dose, and on the anterior and posterior wall mucosal surfaces varied from 128.87% to 138.50% and 142.98% to 180.75% of the prescribed dose. Analysis of 24 actual treatment plans revealed that when the vaginal tissue volume dose covering 98% (vaginal D98%) was comparable between the HZ cylinder and virtual single‐channel plans (6.74 ± 0.07 Gy vs. 6.69 ± 0.10 Gy, *p* = 0.24), rectum doses of HZ cylinder plans were significantly lower than those of single‐channel plans (D1cc, 5.96 ± 0.56 Gy vs. 6.26 ± 0.71 Gy, *p* = 0.02 and D2cc, 5.26 ± 0.52 Gy vs. 5.56 ± 0.62 Gy, *p* = 0.02).

**Conclusions:**

The HZ cylinder demonstrates a reduction in dose to the rectum and bladder while maintaining adequate target volume coverage. Its mucosal surface dose is comparable to that of the traditional single‐channel cylinder. These findings suggest that the HZ cylinder is a viable and potentially safer alternative for vaginal brachytherapy, warranting further investigation with larger sample sizes.

## 1 INTRODUCTION

Endometrial cancer ranks among the most prevalent gynecological malignancies globally, with approximately 417 000 new cases in 2020.^1^ The fundamental approach treating endometrial cancer is surgical procedures, including total hysterectomy, bilateral salpingo‐oophorectomy, and surgical staging. Post‐surgery, treatment may extend to radiotherapy or chemotherapy based on pathological findings.

Vaginal brachytherapy emerges as a crucial postoperative adjuvant therapy for endometrial cancer. Given that 75% of early‐stage endometrial cancer recurrences occur at the upper vagina, studies like PORTEC‐2 have demonstrated that vaginal brachytherapy is as effective as pelvic external radiotherapy for local control in high‐intermediate risk endometrial cancer, but with fewer side effects.^2^ Consequently, vaginal brachytherapy is now the preferred adjuvant treatment for this cancer subtype. Additionally, it plays a significant role as an adjunct to external radiotherapy in treating advanced endometrial cancers with high‐risk factors.^3^, ^4^ While generally considered a straightforward technique, the choice of applicator type and dose fractionation scheme is critical for clinical outcomes.^5^, ^6^

Presently, the single‐channel vaginal cylinder is the most commonly used applicator.^3^, ^7^ However, its single‐channel design limits dose distribution optimization, even with CT simulation imaging. Multi‐channel cylinders offer more flexible dose distribution and can reduce exposure to the rectum and bladder.^8^, ^9^ Yet, a common issue with single‐channel cylinders is the underdosing at the vaginal apex, potentially up to 30%, due to the distance of the radiation source from this region.^10^, ^11^ The clinical significance of this underdose remains a concern.

This study introduces the Hangzhou (HZ) cylinder, a new vaginal brachytherapy applicator designed to address these challenges. The HZ cylinder positions the radioactive source closer to the vaginal apex, ensuring sufficient dose delivery. It also features multiple channels for optimized dose distribution, enhancing protection for the rectum, bladder, and vaginal mucosa. We compare the HZ cylinder dosimetrically with the conventional single‐channel cylinder and validate its clinical application. The study also includes standard plans based on different dose fractionation schemes to assess the equivalent mucosal surface dose.

## 2 METHODS AND MATERIALS

### 2.1 Applicator specifications

**FIGURE 1:**
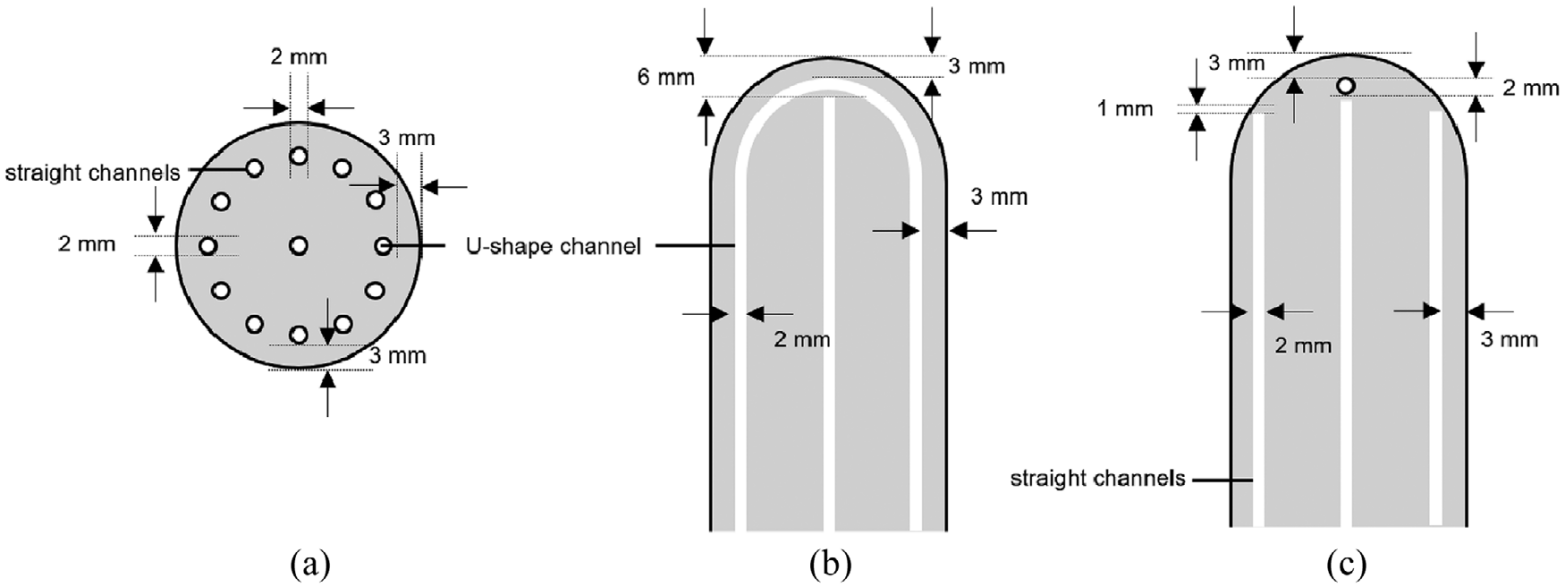
Schematic representation of HZ cylinder. (a) Cross section. (b) U‐shaped channel. (c) Straight channel.

The HZ cylinder is 3D‐printed from C‐UV 9400 resin (C‐UV 9400, Donguan AIDE, China) using a Stereolithography printer (iSLA660, ZRapid Tech, China). It undergoes quality checks, including path patency and medical disinfection. The HZ cylinder consists of a cylinder and a hemisphere, and the hemisphere has the same radius as the cylinder. The top of the hemisphere is fitted with the end of the vagina when used. There are 12 channels of HZ cylinder, and the diameter of all channels is 2 mm. One U‐shaped channel, where the midline is 4 mm from the surface of the HZ cylinder; a central channel, its end is 6 mm from the surface of the hemisphere; 10 straight channels, of which the midlines are 4 mm from the cylinder surface, with their ends are 1 mm from the hemisphere surface (As shown in Figure 1b,c). From the bottom of the HZ cylinder, there are 13 circular holes, right in the middle is the central channel, and the remaining 12 holes are arranged in a pattern resembling the hours 1–12 on a clock face. Among them, two holes of the 3 o'clock and 9 o'clock positions are the U‐shaped channel, as shown in Figure 1a.

**FIGURE 2:**
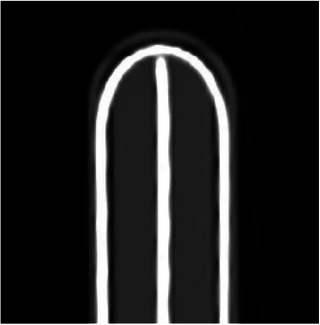
CT scan image of the HZ in coronal section. The highlighted lines are marker wires, which are placed in the U‐shaped channel and the center channel.

A soft plastic tube (KL‐HED‐C, Beijing Kelinzhong Medical Technology Co, Ltd, China), with a 1.98 mm outer diameter and 1.4 mm inner diameter, is compatible with the U‐shaped channel, while soft plastic tubes or metal needles could access the straight channels. Positioned in the vagina, the U‐shaped channel's ends aligned with the 3 o'clock and 9 o'clock positions. Available in 30 and 36 mm diameters, the study used the 30 mm diameter cylinders for both the HZ and single‐channel designs. CT scan image of the HZ cylinder with marker wires is shown in Figure 2.

3D‐printed cylinders with resin filling rates of 100% and 0% were utilized for dose detection, while soft plastic tubes served as the source tubes. When the cylinder was filled with 100% resin, the reading from the well‐type chamber was 31.8 µA. Conversely, when the cylinder was filled with 0% resin, the reading was 32.99 µA, showing a minimal difference of −3.6% between the two measurements. These results suggest that this resin is appropriate for use as applicator material.

### 2.2 Standard plan

A CT simulation scan with 1 mm intervals was conducted, and the channels of both the single‐channel and HZ cylinder applicators (30 mm diameter) were reconstructed by identifying the center of the channels. High‐dose‐rate (HDR) 192 Ir brachytherapy plans were created using the Oncentra system, targeting the cylinder dome and a 3 cm straight part. Dose distribution was optimized to ensure 95% of the prescribed dose at designated points, with attention to minimizing anterior and posterior doses. Plans with different schedules (7 Gy × 3, 5.5 Gy × 4, 5 Gy × 5 fractions) were used for cumulative dose assessment to the vaginal mucosa.

**FIGURE 3:**
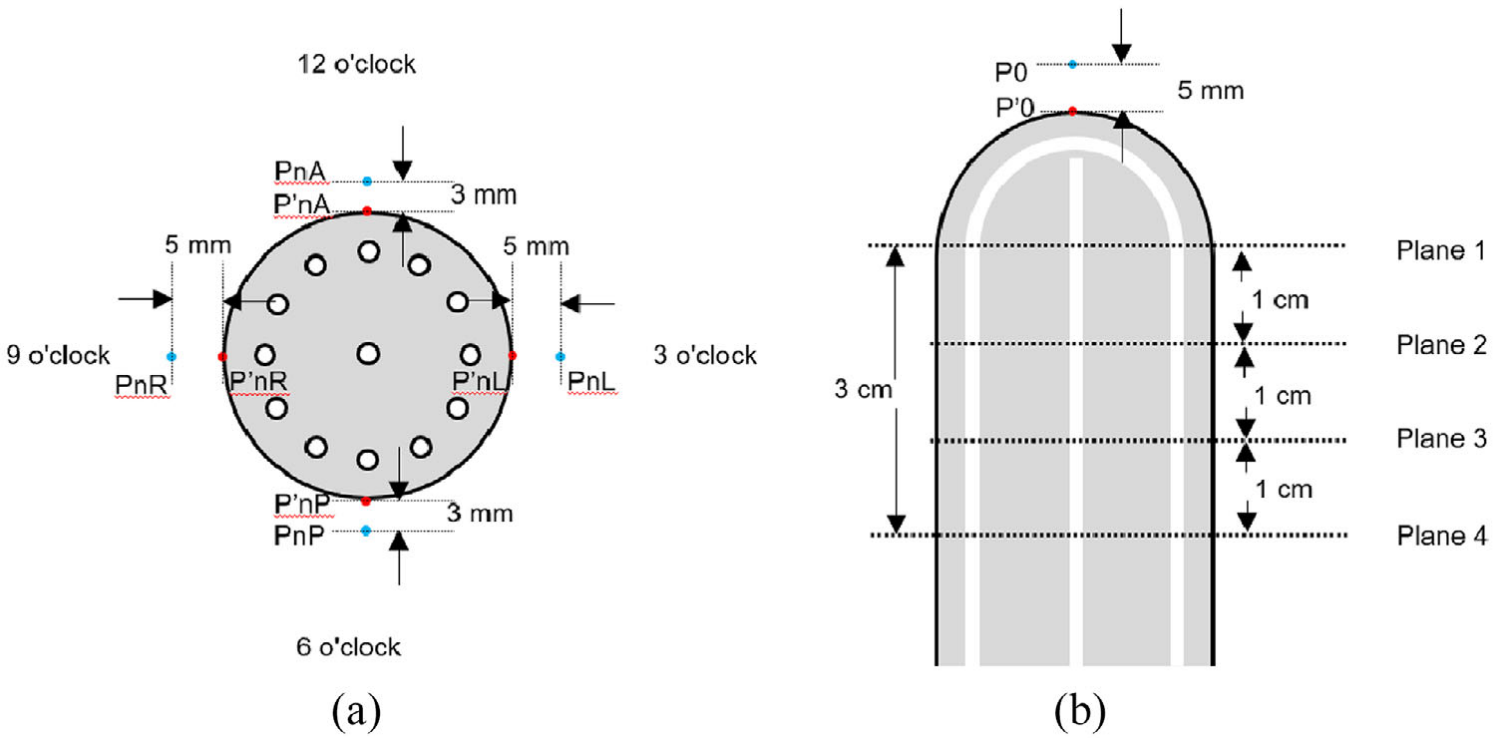
Schematic representation of the 34 dose points, (a) points on each plane, (b) points on the medial axis of the cylinder.

In total, 34 dose points were established, including 17 prescribed dose points and 17 mucosal surface dose points. Prescribed dose points were positioned 5 mm from the mucosal surface along the cylinder's medial axis and on four planes spaced 1 cm apart. P0 was positioned 5 mm from the mucosal surface along the medial axis of the cylinder, PnR and PnL were placed at the 3 and 9 o'clock orientations, 5 mm from the mucosal surface, while PnP and PnA were at the 6 and 12 o'clock orientations, 3 mm from the surface. These points, including their mucosal counterparts, were detailed in Figure 3.

Acknowledging that 95% of lymphatic vessels are located 3 mm below the mucosal surface^12^ and considering the reduced spaces between the vagina‐rectum and vagina‐bladder due to cylinder compression, the prescribed dose points for the HZ cylinder at 12 o'clock and 6 o'clock positions were set at 3 mm below the mucosal surface, instead of the usual 5 mm. This modification aims to enhance protection for both the rectum and bladder.

### 2.3 Treatment plan

Ten patients diagnosed with endometrial cancer underwent post‐surgical vaginal brachytherapy using the HZ cylinder at Zhejiang Cancer Hospital between August 2021 and July 2022 were selected for a retrospective study. These patients were included in this study, which received approval from the Ethics Committee of Zhejiang Cancer Hospital (IRB‐2022‐15) and registered at clinicaltrials.gov (NCT05242861).

Before brachytherapy, patients received enemas and indwelling catheters. Post‐implantation of the HZ cylinder, 50 mL saline was injected into the bladder. A CT simulation was then performed, with 1 mm slice thickness, after placing marker wires in the tubes. The clinical target volume (CTV), including cylinder, was outlined in the Oncentra system, starting 5 mm from the mucosal surface at the vaginal apex, extending 3 cm along the cylinder's straight section, excluding the rectum and bladder. Additional delineations were made for the rectum, bladder, urethra, sigmoid, and small intestine, if present.

Two plans were formulated: one utilizing a U‐shaped channel, a central channel and four straight channels of the HZ cylinder and another using only the central single‐channel for a virtual plan. These plans were shaped by the CTV and OAR volumes, enabling a comparison of dose distribution between the two methods. The main objective was to ensure that 98% of the CTV (CTV D98%) received a 7 Gy dose, while keeping the doses of the OARs as low as possible.

The dose calculation for the treatment plans utilized the TG‐43 formalism, recommended by the American Association of Physicists in Medicine (AAPM).^13^ Dose‐volume histograms (DVHs) were used to calculate the homogeneity index (HI) and conformity index (CI). The evaluation and comparison of all plans were based on several metrics: the dose covering 98% of the vaginal tissue volume (Vagina D98%, with the CTV excluding the applicator) and the volumetric doses for OARs, including D1cc and D2cc for both the bladder and rectum. Definitions for HI and CI were provided as part of the analysis.

$$\mathrm{HI}=\frac{V_{100\%}-V_{150\%}}{V_{\mathrm{vagina}\cdot \mathrm{CTV}}}$$

$$\mathrm{CI}=\frac{V_{\mathrm{CTV}\cdot \mathrm{ref}}}{V_{\mathrm{CTV}}}\times \frac{V_{\mathrm{CTV}\cdot \mathrm{ref}}}{V_{\mathrm{ref}}}$$

where: $V_{100\%}$, the volume of vaginal tissue (CTV excluding the applicator) receiving at least 100% of the prescribed dose. $V_{150\%}$, the volume of vaginal tissue (CTV excluding the applicator) receiving at least 150% of the prescribed dose. $V_{\mathrm{vagina}\cdot \mathrm{CTV}}$, the vaginal tissue volume (CTV excluding applicator). $V_{\mathrm{CTV}\cdot \mathrm{ref}}$, the volume of CTV covered by prescribed reference isodose. $V_{\mathrm{CTV}}$, the volume of total CTV. $V_{\mathrm{ref}}$, the volume of prescribed reference isodose.

### 2.4 Data analysis

The statistical analysis for the study involved the independent samples t‐test for normally distributed data, and the Wilcoxon signed rank test for data not adhering to a normal distribution. Analysis was conducted using the Statistical Package for the Social Sciences (SPSS) version 21, and a *p*‐value of less than 0.05 was considered statistically significant.

## 3 RESULTS

### 3.1 Comparison of point doses between two applicators

Table 1 showed a comparison between the HZ and single‐channel cylinders, with both achieving 95% of the prescribed dose at P0. The HZ cylinder's multi‐channel and U‐shaped design offered superior adjustability. Notably, the point doses toward the vaginal anterior and posterior wall surfaces were significantly lower with the HZ cylinder, especially near the top. This feature maybe crucial for better protection of the rectum and bladder.

**TABLE 1:** Point doses between single‐channel and HZ cylinder.

		*n*% of prescribed dose	
Position	Point	HZ cylinder	Single‐channel cylinder
Apex	P0	95.82	95.70
	P’0	209.32	200.61
Plane1	P1A	100.68	135.15
	P’1A	128.87	180.75
	P1P	100.68	135.15
	P’1P	128.87	180.75
	P1L	97.86	114.10
	P’1L	178.13	180.75
	P1R	97.86	114.10
	P’1R	178.13	180.75
Plane2	P2A	104.09	121.36
	P’2A	129.20	149.80
	P2P	104.09	121.36
	P’2P	129.20	149.80
	P2L	99.80	106.27
	P’2L	149.26	149.80
	P2R	99.80	106.27
	P’2R	149.26	149.80
Plane3	P3A	108.02	115.23
	P’3A	136.93	143.57
	P3P	108.02	115.23
	P’3P	136.93	143.57
	P3L	99.52	100.82
	P’3L	152.99	143.57
	P3R	99.52	100.82
	P’3R	152.99	143.57
Plane4	P4A	105.80	110.99
	P’4A	138.50	142.98
	P4P	105.80	110.99
	P’4P	138.50	142.98
	P4L	95.16	95.66
	P’4L	153.98	142.98
	P4R	95.16	95.66
	P’4R	153.98	142.98

### 3.2 Mucosal surface dose under different fractionation schedules

Three dose fractionation schemes (7 Gy×3, 5.5 Gy×4, and 5 Gy×5) were compared. The total prescribed bioequivalent dose for the 2 Gy per fraction, α/β = 10 (EQD2_10_) was similar in these regimens. We compared the HZ cylinder with the single‐channel cylinder currently in use, along with two non‐MRI‐compatible single‐channel cylinders and one MRI‐compatible single‐channel applicator from the literature.^14^, ^15^, ^16^ All applicators had a diameter of 3 cm. Table 2 listed EQD2_10_ for the vaginal apical and lateral wall surfaces, comparing the results of this study with previous studies. At these prescriptions, vaginal mucosal doses from the HZ cylinder were consistent with those from other applicators. Notably, the 5.5 Gy×4 regimen showed the lowest EQD2_10_ at the mucosal surface.

**TABLE 2:** The mucosal surface EQD2_10_ of three dose fractionation schemes.

Dose fractionation scheme	Applicator	Surface to closest dwell point (cm)^a^	Apical surface EQD2_10_ per fx (Gy)	Lateral surface EQD2_10_ per fx (Gy)	Total apical surface EQD2_10_ (Gy)	Total lateral surface EQD2_10_ (Gy)
7 Gy×3	HZ cylinder	0.4	32.23	20.17	96.69	60.50
	Single‐channel cylinder	1.1	30.17	17.49	90.51	52.46
	Single‐channel cylinder^14^	–	19.25	–	57.75	–
	CT‐MR single‐channel cylinder^15^	0.8	32.12	19.79	96.37	59.36
	Single‐channel cylinder^16^	–	26.68	19.98	80.05	59.93
5.5 Gy×4	HZ cylinder	0.4	22.04	14.04	88.17	56.16
	Single‐channel cylinder	1.1	20.68	12.24	82.74	48.98
	Single‐channel cylinder^14^	–	13.56	–	54.23	–
	CT‐MR single‐channel cylinder^15^	0.8	21.97	13.79	87.89	55.15
	Single‐channel cylinder^16^	–	18.40	13.91	73.52	55.65
5 Gy×5	HZ cylinder	0.4	19.04	12.22	95.22	61.09
	Single‐channel cylinder	1.1	17.89	10.68	89.44	53.39
	Single‐channel cylinder^14^	–	11.78	–	58.92	–
	CT‐MR single‐channel cylinder^15^	0.8	18.98	12	94.92	60
	Single‐channel cylinder^16^	–	15.9	12.11	79.62	60.54

^a^ The distance from the surface of the cylinder apex to the nearest dwell position of the radiation source. Data not available.

### 3.3 Treatment plans of two applicators

**FIGURE 4:**
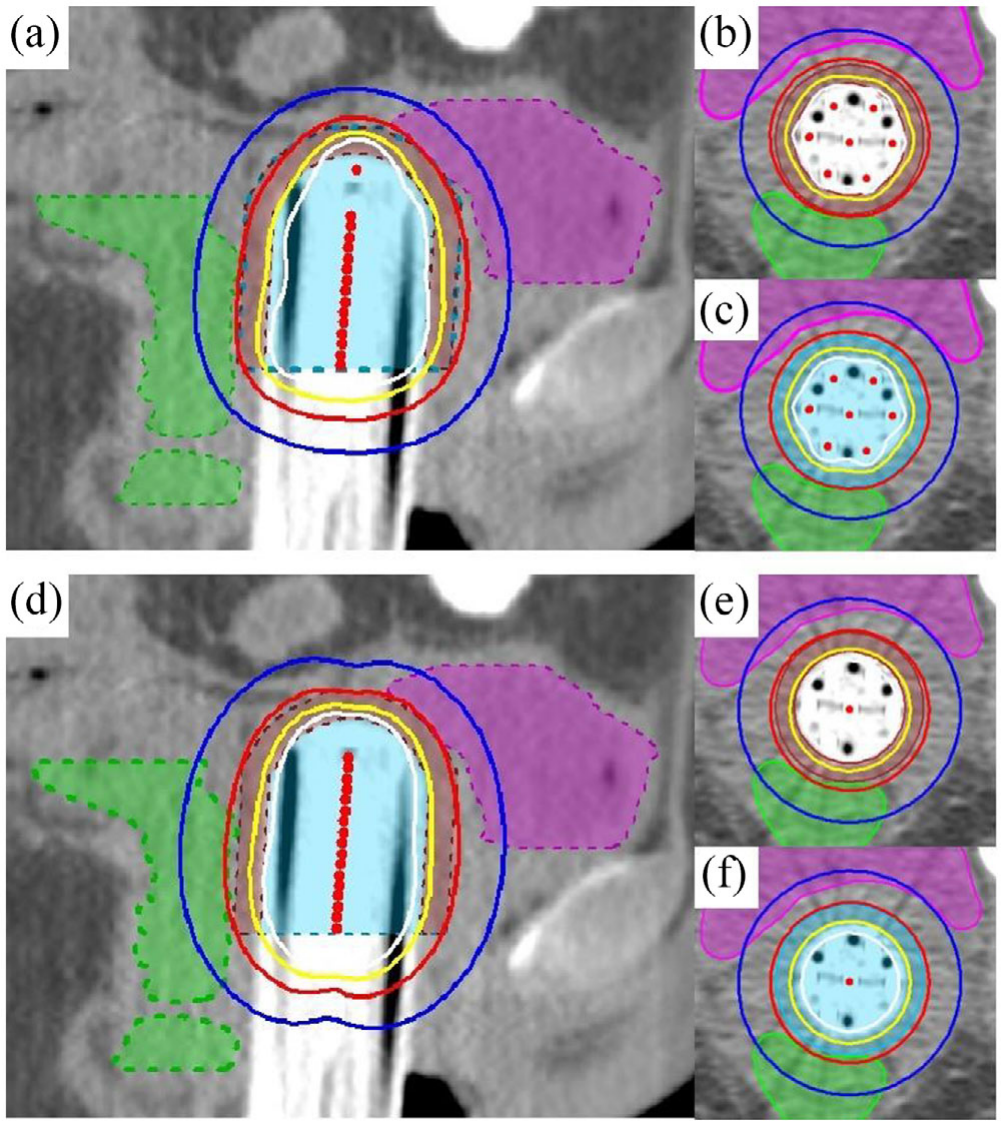
Dose distribution comparison between HZ cylinder (A) and single‐channel (D) in sagittal views, vaginal tissue volume shown in HZ cylinder (B) and single‐channel (E) in axial views, clinical target volume shown in HZ cylinder (C) and single‐channel (F) in axial views. Blue shaded contour = clinical target volume (CTV), brown shaded contour = vaginal tissue volume (CTV excluding the applicator), magenta shaded contour = bladder, green shaded contour = rectum. Isodose line of 7 Gy (prescribed dose) is displayed in red, 10.5 Gy (150% of prescribed dose) in yellow, 14 Gy (200% of prescribed dose) in white.

Data from 10 patients who received CT‐guided vaginal cuff HDR brachytherapy using the HZ cylinder were retrospectively reviewed. A total of 24 HDR brachytherapy plans were executed and analyzed. For each patient, dose distributions and DVHs for vagina (CTV minus cylinder) and OAR were generated for both HZ cylinder plans and virtual single‐channel plans. Figure 4 in the study presented a comparative example of dose distributions from the two plans for a representative patient. Table 3 summarized the dosimetric comparison between the single‐channel and HZ cylinder plans.

**TABLE 3:** Dosimetric comparison between single‐channel and HZ cylinder, presented as mean and standard deviation for fractional prescribed dose of 7 Gy.

	VaginaD98% (Gy)	HI	CI	BladderD1cc (Gy)	BladderD2cc (Gy)	RectumD1cc (Gy)	RectumD2cc (Gy)
HZ cylinder	6.74 ± 0.07	0.69 ± 0.02	0.79 ± 0.03	6.11 ± 0.66	5.70 ± 0.63	5.96 ± 0.56	5.26 ± 0.52
Single‐channel	6.69 ± 0.10	0.76 ± 0.28	0.76 ± 0.22	6.23 ± 0.53	5.82 ± 0.51	6.26 ± 0.71	5.56 ± 0.62
*p*	0.24	0.00	0.02	0.60	0.60	0.02	0.02

The DVH analysis showed that the vaginal tissue D98% for the HZ cylinder plans was slightly higher than the single‐channel plans, but without a statistical difference. The homogeneity index (HI) of the vaginal tissue in single‐channel plans was significantly higher than that of the HZ cylinder plans (*p* < 0.01). Additionally, there was a notable difference in the conformity index (CI) for the CTV between the two applicators (*p* = 0.02), with the HZ cylinder showing a higher CI.

The DVH analysis indicated that the rectal dose in single‐channel plans was statistically higher than in HZ cylinder plans (6.26 ± 0.71 Gy vs. 5.96 ± 0.56 Gy for rectal D1cc and 5.56 ± 0.62 Gy vs. 5.26 ± 0.52 Gy for rectal D2cc, with *p* = 0.02 for both). However, for bladder doses, the DVH showed no statistical difference in D1cc and D2cc between HZ cylinder plans and single‐channel plans.

## 4 DISCUSSION

Postoperative vaginal brachytherapy for endometrial cancer typically targets a 5 mm depth under the mucosal surface, aligning with the fact that 50% of lymphatic vessels are located 1 mm below the vaginal mucosa, while 95% are within 3 mm depth.^12^ However, standard commercial cylinders may underdose the vaginal apex by up to 30%.^10^, ^11^ Moreover, Palmgren et al.^17^ determined through CT simulation that to cover 82% of the vaginal apical scar tissue, the prescription point should be placed 10 mm below the apical mucosa, further illustrates the challenge of achieving sufficient dose coverage at the submucosal level of the vaginal apex. It highlights the difficulty of adequately treating the vaginal apex using conventional methods. This highlights the challenge of adequately treating the vaginal apex with conventional methods. In this study, the HZ cylinder was innovatively designed with a U‐shaped channel, positioning the radioactive source just 4 mm from the dome surface, improving dose coverage in this critical area.

In general, the total dose of vaginal brachytherapy alone after surgery for endometrial cancer is not high, regardless of applicator type and treatment regimen. However, because early‐stage endometrial cancer has a longer survival period, the protection of normal tissue is very important for quality of life. The dose on the mucosal surface of the vaginal apex was 209.32% of the prescribed dose, on the left and right sides were 149.26% to 178.13% of the prescribed dose, and on the anterior and posterior sides were 128.87% to 138.50% of the prescribed dose when using HZ cylinder. The isodose lines in the anterior‐posterior direction were retracted to protect OARs. In terms of treatment plan, when the CTV D98% reached 7 Gy, the D98% of the vaginal tissue of the HZ cylinder was slightly higher than that of the single‐channel, and the rectal dose was significantly lower than that of the single channel. This demonstrated the HZ cylinder's efficacy in balancing target dose coverage and OAR protection.

Determining the optimal dose for the vaginal mucosal surface in brachytherapy remains unresolved^18^, ^19^ Theoretically, a lower dose ratio of the dose at mucosal surface P’ to the dose at 5 mm depth under the mucosal surface P(RP’/P) is preferable, indicating less dose at the mucosal surface. According to the dose decay characteristics of brachytherapy, the further away from the radioactive source, the lower of RP’/P. However, keeping the radiation source away from the apex of the vagina in order for a low RP’/P will result in an increased dose in the anterior and posterior directions, which increases the dose to the bladder and rectum. Therefore, the balance between RP’/P and the distance from the radioactive source to the cylinder surface is very important. Previous studies reported RP’/P values ranging from 155% to 218% for single‐channel cylinder.^14^, ^15^, ^16^ In this study, the HZ cylinder showed a balanced dosimetry, slightly higher RP’/P at the apex and sidewalls, and lower in the anterior and posterior directions compared to the single‐channel cylinder, indicating effective target volume coverage and mucosal protection.

The HZ cylinder effectively balances target volume coverage and OAR protection due to two key factors: Firstly, its design allows dwell points to be as close as 4 mm from the vaginal apical mucosal surface, ensuring adequate coverage without excessive dosing in other directions. Secondly, multiple dwell points, including those in the U‐shaped channel and at the top of other 10 channels, facilitates a uniform dose distribution, keeping the dose to the vaginal surface within a safe range.

There are many different brachytherapy schedules in use. All of these schedules have reported excellent efficacy and low toxicity.^3^, ^18^, ^20^ Although the schedule of 7 Gy (at 5 mm depth under mucosal surface)×3 from PORTEC‐2 is the most widely accepted,^2^, ^7^ the optimal schedule remains unclear. In this study, the total vaginal mucosal surface dose was compared between the HZ cylinder and other cylinders under different treatment schedules by calculating the bioequivalent dose. With a 5.5 Gy×4 schedule, the total EQD2_10_ of 88.17 Gy and 56.16 Gy for vaginal apical and left/right side wall surface respectively were the lowest. Considering the late toxicity of the vaginal mucosa, the 5.5 Gy×4 schedule may be most appropriate for the HZ cylinder. Other schedules, however, were also reasonable, as the total EQD2_10_ of vaginal apex provided by HZ cylinder were not high.

3D printing technology for making applicators has shown effectiveness in dose optimization.^21^, ^22^, ^23^ There are two approaches: creating individualized shapes and channels,^24^, ^25^ and designing channels in a standard cylinder.^26^ The HZ cylinder uses the latter, offering suitability for most patients while saving on time and costs associated with personalized applicators. However, its minimum diameter of 30 mm limits its use in patients with a narrow vagina, as cylinders with a smaller diameter require a sharper curvature, which may affect the passage of the radiation source, indicating a need for further research to optimize dose distribution for such cases.

This study presents several limitations. Firstly, in comparing standard plans, it is assumed that the radioactive source travels along the center line of the soft plastic tube, disregarding its actual path through the curved tube. However, given the close fit between the tube's outer wall (1.98 mm diameter) and the channel's inner wall (2 mm diameter), and considering the tube's inner diameter (1.4 mm) and the radioactive source's outer diameter (0.9 mm), the resulting error is minimal. Additionally, the patient's treatment plan is formulated using a marker wire to reconstruct the actual path, further mitigating this issue. Secondly, the study did not evaluate different after‐loading machines from various manufacturers, particularly in the context of the tube's curvature at the U‐shape. This raises uncertainty about the applicability of our findings to other after‐loading machines. Thirdly, the sample size of 10 patients is relatively small, and future studies with larger sample sizes are needed to confirm these findings. Moreover, due to the unavailability of a 3 cm diameter multi‐channel commercial cylinder, we could not compare the HZ cylinder with a commercially available multi‐channel cylinder, which is another limitation of this study.

## 5 CONCLUSION

The study has demonstrated that the HZ cylinder is a feasible and effective option for adjuvant brachytherapy following hysterectomy. It effectively balances adequate target volume coverage and the protection of organs at risk, including the vaginal mucosa.

## AUTHOR CONTRIBUTIONS

**Xiang Zhang**: Conceptualization; data curation; formal analysis; methodology; writing—original draft; writing—review & editing. **Yanfei Lu**: Data curation; formal analysis; writing—original draft; writing—review & editing. **Jianhong Chen**: Conceptualization; data curation; formal analysis; methodology. **Fangfang Wang**: Conceptualization; data curation; formal analysis; methodology. **Qiong Zhou**: Conceptualization; data curation; formal analysis; methodology.

## CONFLICT OF INTEREST STATEMENT

The authors declare no conflicts of interest.

## CLINICAL TRIAL INFORMATION

This research has received approval from the Ethics Committee of Zhejiang Cancer Hospital (IRB‐2022‐15) and registered at clinicaltrials.gov (NCT05242861).

## Data Availability

Research data are not shared.

## References

[1] Sung H , Ferlay J , Siegel RL , et al. Global cancer statistics 2020: GLOBOCAN estimates of incidence and mortality worldwide for 36 cancers in 185 countries. CA Cancer J Clin. 2021;71(3):209‐249. 33538338 10.3322/caac.21660

[2] Nout RA , Smit V , Putter H , et al. Vaginal brachytherapy versus pelvic external beam radiotherapy for patients with endometrial cancer of high‐intermediate risk (PORTEC‐2): an open‐label, non‐inferiority, randomised trial. Lancet. 2010;375(9717):816‐823. 20206777 10.1016/S0140-6736(09)62163-2

[3] Small W , Beriwal S , Demanes DJ , et al. American brachytherapy society consensus guidelines for adjuvant vaginal cuff brachytherapy after hysterectomy. Brachytherapy. 2012;11(1):58‐67. 22265439 10.1016/j.brachy.2011.08.005

[4] Paulson K , Logie N , Han G , et al. Adjuvant radiotherapy in stage II endometrial cancer: selective de‐intensification of adjuvant treatment. Clin Oncol J R Coll Radiol. 2023(1):e94‐e102. 10.1016/j.clon.2022.08.034 36150980

[5] Sabater S , Andres I , Lopez‐Honrubia V , et al. Vaginal cuff brachytherapy in endometrial cancer—a technically easy treatment? Cancer Manag Res. 2017;9:351‐362. 28848362 10.2147/CMAR.S119125 PMC5557121

[6] Soror T , Chafii R , Lancellotta V , Tagliaferri L , Kovács G . Biological planning of radiation dose based on in vivo dosimetry for postoperative vaginal‐cuff HDR interventional radiotherapy (Brachytherapy). Biomedicines. 2021;9(11). 10.3390/biomedicines9111629 PMC8615499 34829858

[7] Harkenrider MM , Block AM , Alektiar KM , et al. American brachytherapy task group report: adjuvant vaginal brachytherapy for early‐stage endometrial cancer: a comprehensive review. Brachytherapy. 2017;16(1):95‐108. 27260082 10.1016/j.brachy.2016.04.005 PMC5612425

[8] Iftimia I , Cirino ET , Mower HW , Mckee AB . Treatment planning methodology for the miami multichannel applicator following the American Brachytherapy Society recently published guidelines: the Lahey Clinic experience. J Appl Clin Med Phys. 2013;14(1):4098. 23318396 10.1120/jacmp.v14i1.4098 PMC5714050

[9] Abtahi M , Safaei AM , Sheikhzade P , Gholami S . A Dosimetric evaluation of a high dose rate cobalt‐60 brachytherapy source using shielded, single and multi‐channel cylinder applicators for gynecological cancers. Med Dosim. 2022;47(4):318‐324. 35907692 10.1016/j.meddos.2022.06.001

[10] Li Z , Liu C , Palta JR . Optimized dose distribution of a high dose rate vaginal cylinder. Int J Radiat Oncol Biol Phys. 1998;41(1):239‐244. 9588940 10.1016/s0360-3016(98)00014-5

[11] Gore E , Gillin MT , Albano K , Erickson B . Comparison of high dose‐rate and low dose‐rate dose distributions for vaginal cylinders. Int J Radiat Oncol Biol Phys. 1995;31(1):165‐170. 7995748 10.1016/0360-3016(94)00326-G

[12] Choo JJ , Scudiere J , Bitterman P , Dickler A , Gown AM , Zusag TW . Vaginal lymphatic channel location and its implication for intracavitary brachytherapy radiation treatment. Brachytherapy. 2005;4(3):236‐240. 16182225 10.1016/j.brachy.2005.02.002

[13] Rivard MJ , Coursey BM , Dewerd LA , et al. Update of AAPM task group no. 43 report: a revised AAPM protocol for brachytherapy dose calculations. Med Phys. 2004;31(3):663‐674. 10.1118/1.1646040 15070264

[14] Albuquerque K , Hrycushko BA , Harkenrider MM , et al. Compendium of fractionation choices for gynecologic HDR brachytherapy − an American Brachytherapy Society Task Group Report. Brachytherapy. 2019;18(4):429‐436. 30979631 10.1016/j.brachy.2019.02.008

[15] Li S , Aref I , Walker E , Movsas B . Effects of prescription depth, cylinder size, treatment length, tip space, and curved end on doses in high‐dose‐rate vaginal brachytherapy. Int J Radiat Oncol Biol Phys. 2007;67(4):1268‐1277. 17336226 10.1016/j.ijrobp.2006.10.041

[16] Kim RY , Pareek P , Duan J , Murshed H , Brezovich I . Postoperative intravaginal brachytherapy for endometrial cancer; dosimetric analysis of vaginal colpostats and cylinder applicators. Brachytherapy. 2002;1(3):138‐144. 15090276 10.1016/s1538-4721(02)00051-x

[17] Palmgren J‐E , Seppälä J , Anttila M . Is adaptive treatment planning for single‐channel vaginal brachytherapy necessary?. J Contemp Brachyther. 2021;13(6):687‐693. 10.5114/jcb.2021.112120 PMC8782068 35079256

[18] Delishaj D , Barcellini A , D'amico R , et al. Vaginal toxicity after high‐dose‐rate endovaginal brachytherapy: 20 years of results. J Contemp Brachythe *r*. 2018;10(6):559‐566. 10.5114/jcb.2018.79713 PMC6335557 30662479

[19] Lancellotta V , Macchia G , Dinapoli N , et al. EROS 2.0 study: evaluation of two interventional radiotherapy (brachytherapy) schedules for endometrial cancer: a comparison of late vaginal toxicity rates. Radiol Med (Torino). 2022;127(3):341‐348. 35092552 10.1007/s11547-022-01455-y PMC8960610

[20] Arden JD , Dokter J , Almahariq MF , et al. Toxicity and efficacy after adjuvant vaginal brachytherapy using 30 Gy in 6 fractions for stages I and II endometrial cancer. Adv Radiat Oncol. 2021;6(6):100773. 34934859 10.1016/j.adro.2021.100773 PMC8655421

[21] Wiebe E , Easton H , Thomas G , Barbera L , D'alimonte L , Ravi A . Customized vaginal vault brachytherapy with computed tomography imaging‐derived applicator prototyping. Brachytherapy. 2015;14(3):380‐384. 25630618 10.1016/j.brachy.2014.12.006

[22] Sekii S , Tsujino K , Kosaka K , et al. Inversely designed, 3D‐printed personalized template‐guided interstitial brachytherapy for vaginal tumors. J Contemp Brachyther. 2018;10(5):470‐477. 10.5114/jcb.2018.78832 PMC6251441 30479625

[23] Fahimian BP , Liu WU , Skinner L , et al. 3D printing in brachytherapy: a systematic review of gynecological applications. Brachytherapy. 2023(4):446‐460. 37024350 10.1016/j.brachy.2023.02.002

[24] Paterson DB , Pearson SM , Wilson AN . Intracavitary vaginal brachytherapy using a custom balloon applicator. J Med Radiat Sci. 2017;64(4):310‐314. 28661036 10.1002/jmrs.235 PMC5715253

[25] Yan J , Qin X , Zhang F , Hou X , Yu L , Qiu J . Comparing multichannel cylinder and 3D‐printed applicators for vaginal cuff brachytherapy with preliminary exploration of post‐hysterectomy vaginal morphology. J Contemp Brachyther. 2021;13(6):641‐648. 10.5114/jcb.2021.112115 PMC8782066 35079250

[26] Ricotti R , Vavassori A , Bazani A , et al. 3D‐printed applicators for high dose rate brachytherapy: dosimetric assessment at different infill percentage. Phys Med. 2016;32(12):1698‐1706. 27592531 10.1016/j.ejmp.2016.08.016

